# The role of TMEM59L in colorectal cancer progression and its interaction with the TGF-β/Smad pathway

**DOI:** 10.3389/fonc.2025.1674849

**Published:** 2025-11-25

**Authors:** Hongjie Yang, Jiafei Liu, Peishi Jiang, Yi Sun, Lingyi Chen, Siwei Zhu

**Affiliations:** 1 School of Medicine, Nankai University, Tianjin, China; 2 Department of Colorectal Surgery, Tianjin Union Medical Center, The First Affiliated Hospital of Nankai University, Tianjin, China; 3 The Institute of Translational Medicine, Tianjin Union Medical Center of Nankai University, Tianjin, China; 4 Tianjin Institute of Coloproctology, Tianjin, China; 5 State Key Laboratory of Medicinal Chemical Biology, Tianjin Key Laboratory of Protein Sciences, Frontiers Science Center for Cell Responses, National Demonstration Center for Experimental Biology Education and College of Life Sciences, Nankai University, Tianjin, China; 6 Department of Oncology, Tianjin Union Medical Center, The First Affiliated Hospital of Nankai University, Tianjin, China

**Keywords:** colorectal neoplasms, TMEM59L, TGF-β signaling pathway, metastasis, therapeutic target

## Abstract

**Objective:**

This study aimed to investigate the role of TMEM59L in colorectal cancer (CRC) and its interaction with the TGF-β/Smad signaling pathway.

**Methods:**

We analyzed the correlation between TMEM59L expression levels and patient survival, as well as its impact on the TGF-β/Smad signaling pathway, using data from The Cancer Genome Atlas (TCGA). Additionally, transwell, CCK-8, EdU, and colony formation assays were conducted to assess the effects of TMEM59L on CRC cell migration, invasion, and proliferation. Gene silencing and overexpression, along with specific inhibitors/agonists, were used to validate the involvement of TMEM59L in the regulation of the TGF-β/Smad signaling pathway.

**Results:**

We found that high TMEM59L expression was associated with poor patient survival and TGF-β pathway activation. After si-TMEM59L treatment, the migration and invasion abilities of CRC cells were reduced, while cell proliferation remained affected to a lesser extent. Additionally, the levels of TGF-β protein were decreased, and the phosphorylation of Smad2/3 was reduced. *In vivo*, TMEM59L knockdown reduced metastatic potential as demonstrated by decreased fluorescence intensity, while overexpression of TMEM59L increased metastatic potential, which was reversed by TGF-β inhibition.

**Conclusion:**

TMEM59L may promote CRC metastasis by enhancing cell migration and invasion, with minimal impact on cell proliferation, potentially through the TGF-β/Smad signaling pathway.

## Introduction

Colorectal cancer (CRC) is one of the most common digestive system cancers worldwide, accounting for 10.0% of all cancer cases (1). As the burden of colorectal cancer increases, the identification of effective targeted therapy targets has become increasingly important. TMEM (Transmembrane proteins) are widely distributed proteins in cell membranes with multiple transmembrane domains. They play a key role in tumor progression, influencing processes like cell proliferation, migration, and invasion (2, 3). TMEM45A (4), TMEM45B (5), TMEM48 (6), TMEM98 (7), TMEM119 (8), and TMEM168 (9), proteins are considered oncogenes in certain types of cancer because they contribute to increased cell proliferation, migration, invasion, and EMT. In contrast, proteins such as TMEM106A (10), TMEM170B (11), and TMEM88 (12) are considered tumor suppressors, as they reduce cell proliferation, migration, invasion, and EMT in some cancer types.

TMEM59L was first discovered in 1999 and was initially considered a protein expressed specifically in the brain (13). Research on the mechanism of this protein has primarily focused on the nervous system (14–16). In 2022, the link between TMEM59L and cancers was first discovered. Since then, numerous studies have found that TMEM59L is associated with the survival of patients with cancers (17–19). However, these studies are primarily based on bioinformatics analyses from databases, and there is currently a lack of cell and animal experimental research. Bioinformatics studies have reported that TMEM59L is associated with the activation of the TGF-β/signaling pathway (17). This study aims to explore the effects of TMEM59L on CRC tumor cells and its relationship with the TGF-β signaling pathway through cell and animal experiments. To our knowledge, this is the first study to systematically validate the function of TMEM59L in colorectal cancer through comprehensive *in vitro* and *in vivo* experiments, providing a theoretical foundation for developing novel anti-metastatic therapeutic strategies.

## Materials and methods

### Bioinformatics analysis

The RNA-seq gene expression data and corresponding clinical information of colorectal cancer patients were obtained from The Cancer Genome Atlas (TCGA) database, and the transcriptional levels of TMEM59L mRNA were extracted. Using the optimal cutoff algorithm, the cutoff point that minimized the Log-rank test p-value was determined to classify patients into high and low TMEM59L expression groups. Kaplan-Meier survival curves were then plotted to assess the relationship between TMEM59L expression levels and patient survival rates. Additionally, gene set enrichment analysis (GSEA) was performed to examine whether gene expression in the high TMEM59L expression group was significantly enriched in TGF-β-related signaling pathways. To further investigate the clinical relevance of TMEM59L, we analyzed its expression distribution across pathological stages (I-IV) using violin plots with trend analysis. Multivariable Cox regression analysis was performed using a stepwise approach, progressively adjusting for age, gender, and pathological stage to evaluate the independent prognostic value of TMEM59L expression.

### Cell lines and cell culture

The human colorectal cancer cell lines LoVo and HCT116 were obtained from ATCC. The human colorectal cancer cell line HCT116-luc and four derived stable cell lines were obtained from Shanghai Zhong qiao xin zhou biotechnology. The stable cell lines included: TMEM59L knockdown cells (HCT116-luc#hTMEM59L[shRNA], referred to as TMEM59L_KD) with corresponding scramble shRNA control (HCT116-luc#Scramble_shRNA, referred to as Control_KD), and TMEM59L overexpression cells (HCT116-luc#hTMEM59L, referred to as TMEM59L_OE) with corresponding vector control (HCT116-luc#Control, referred to as Control_OE). These cell lines were generated using the Neon™ transfection system (Cat# MPK5000) and selected using hygromycin resistance. All cell lines were validated by quantitative PCR using specific primers for TMEM59L and GAPDH as internal control. The experimental operation was carried out in strict accordance with the instructions on the official website. The supernatant was removed by digestion and centrifugation of the target cells growing logarithmically in good condition (suspended cells could be collected directly), and the cell precipitate was obtained by re-suspension counting with PBS and 1×10^6^ cells were taken into the EP tube for centrifugation. The cells were suspended with electrolysis buffer, and the plasmids were added and thoroughly mixed. Input the optimal power parameters, power the above mixture, and power the EGFP control group under the same parameters. The cells in each group after electrotransfer were transferred to the corresponding culture vessel and cultured in the incubator, and the cell status was observed after 24h, and the electrotransfer efficiency was judged according to the EGFP control group. 48h after cell culture, the target cells after electrotransfer were screened with the screening drug corresponding to the scheme. After the drug screening cycle, the drug-containing medium was removed and replaced with fresh medium, and the cell state was restored after culture for 24-48h.) Cell lines were maintained in RPMI-1640 medium (Invitrogen, Carlsbad, CA, USA) supplemented with 10% fetal calf serum (Invitrogen). Cells were cultured at 37 °C with 5% CO_2_. Antibiotics are purchased from shanghai yesen biotechnology.

### *In vivo* metastasis experiments

Animal study was approved by the independent ethics committee of Tianjin jinke biotechnology. Nude mice (HFK Bio-Technology, Beijing, China) were maintained in accordance with the ethics standards of the World Medical Association (Declaration of Helsinki). Cells were injected to the caudal vein of 16–19 g female nude mice as a 100 µL suspension (5 × 10^5^ cells). After 28 days, mice were sacrificed and lungs were stripped for analysis. D-luciferin potassium salt D (40902ES01) was purchased from shanghai Yesen Biotechnology (Shanghai, China). Bioluminescence imaging was performed in the afternoon on days 0, 7, 14, 21, and 28 post-cell injection. For imaging, potassium luciferin was administered at a dose of 150 mg/kg by intraperitoneal injection. The potassium luciferin was fully dissolved in sterile PBS without Mg2+ and Ca2+, and filtered through a 0.22-micron microporous membrane. Imaging was performed at the peak fluorescence intensity, 10 minutes after the luciferin injection. The TGF-β pathway inhibitor SB-431542 was purchased from Yesen Biotechnology (Shanghai, China) and administered at a dose of 10 mg/kg on days 7, 14, 21, and 28 post-cell injection. It was fully dissolved in 5% DMSO and administered by intraperitoneal injection in the morning, prior to the afternoon imaging sessions.

### Hematoxylin-eosin staining

Lungs were immersed in 4% paraformaldehyde for 24 h and transferred to 60% ethanol. Individual lobes of lung biopsy material were placed in processing cassettes, dehydrated through the serial alcohol gradients, and embedded in paraffin. Before immunostaining, 5 μm thick lung tissue sections were dewaxed in xylene, rehydrated through decreasing concentrations of ethanol, and washed in PBS. Then sections were stained with hematoxylin and eosin. After staining, sections were dehydrated through increasing concentrations of ethanol and xylene.

### Proliferation assay

Primary cells were seeded in 96-well plates and treated as needed. Cell viability was measured 24\48\72\96 hours after treatment cessation using the Cell-Counting Kit 8 (CCK-8) (40203ES80) from shanghai yesen biotechnology. Plates were incubated for 1.5 hours at 37°C and absorbance was measured at 450nm using the Infinite 200 Tecan i-control plate reader machine. The CCK-8 assay was performed in at least 3 replicates for each experimental condition.

Cells were seeded in a confocal fluorescence microscope special dish and treated with siTMEM59L or NC for 48 h. Cells proliferation was tested using EdU Cell Proliferation Kit (40276ES76) from shanghai yesen biotechnology with Alexa Fluor 594 in accordance with the operating instructions for processing. Cell nuclei were co-stained with Hoechst 33258. After that, EdU-positive cells were monitored with a fluorescence microscope (Olympus Corporation, Tokyo, Japan).

To observe the capacity changes of single cells to form a colony, cells were inoculated treated with siTMEM59L or NC for 48 h. The fresh RPMI1640 medium was used every 3 days to replace the medicated culture medium, and cells were cultured for 10 days. Then, the cellular colonies were immobilized, treated with crystal violet solution and the numbers of colonies were counted.

### Cell migration and invasion assays

Cell migration assay was performed in 24-well CIM plates (BD Biosciences, CA, USA). Briefly, 1-3 × 10^4^ cells per well were seeded in serum-free medium in the upper compartment of the CIM plates. Serum-complemented medium was added to the lower compartment of the chamber. After 24 h incubation, cells that passed through the septum were fixed with cold methanol and stained with crystal violet. The average number of migrated cells in four random microscopic fields was counted. Cell invasion assay was performed in 24-well CIM plates coated with matrigel. Other steps were identical to those of cell migration assay.

### Western blot analysis

Cells were homogenized in loading buffer (0.1 M Tris-HCl, pH 6.8, 1% SDS, 10% β-mercaptoethanol, 11% glycerol), separated by SDS-PAGE and electro-blotted to the nitrocellulose membranes, then blocked with 5% non-fat milk in TBS for 1.5 h at room temperature. The membranes were incubated with the indicated primary antibodies at 4 °C overnight. After washing three times with TBST, membranes were probed by horseradish peroxidase-labeled secondary antibodies for 45 min at room temperature. Protein bands were visualized by enhanced chemiluminescence. Anti-GAPDH (60004) was from Proteintech (Chicago, IL, USA). The secondary antibodies were purchased from Abcam (Cambridge, UK). Phospho-Smad2/3 (Thr8) (31132ES50), Phospho-Smad2 (Ser467) (31131ES50), Smad2 (31058ES50), TGF-β1 (30013ES50) was purchased from Yeasen Biotechnology (Shanghai, China). TMEM59L (PA5-72688) was purchased from Invitrogen (Thermo Fisher Scientific, Waltham, MA, USA).

### RNA interference

Small interfering RNAs (siRNAs) were provided by GenePharma (Shanghai, China) and transfected into cells with siRNA Mate (GenePharma) following the provider’s instructions.

Interference sequences used were:

TMEM59L #1 -sense:
5’- GCGUGGAAGCCUAUGUGAATT -3’;
TMEM59L #1 -antisense:
5’- UUCACAUAGGCUUCCACGCTT-3’;
TMEM59L #2 -sense:
5’- GCAAUGACCUUGUCAACUCTT -3’;
TMEM59L #2 -antisense:
5’- GAGUUGACAAGGUCAUUGCTT -3’;
TMEM59L #3 -sense:
5’- GGCCAAGGUGGAGUCUGAATT -3’;
TMEM59L #3 -antisense:
5’- UUCAGACUCCACCUUGGCCTT -3’;
TMEM59L #4 -sense:
5’- GCACAAGGGCUUCAUGAUGTT -3’;
TMEM59L #4 -antisense:
5’- CAUCAUGAAGCCCUUGUGCTT -3’;
Negative control -sense:
5’- UUCUCCGAACGUGUCACGUTT -3’;
Negative control -antisense:
5’ -ACGUGACACGUUCGGAGAATT -3’.

### Statistical analysis

Bioinformatics analysis was performed using R (version 4.3.3), and other statistical analyses were done using Python (version 3.9.7). The unpaired two-tailed t-test was used for *in vitro* study and vivo study. A two-sided p < 0.05 was considered statistically significant. *** p < 0.001; ** p < 0.01; * p < 0.05; N.S. p > 0.05.

## Result

### High TMEM59L expression predicts poor survival and is linked to TGF-β pathway enrichment in colorectal cancer

**Figure 1:**
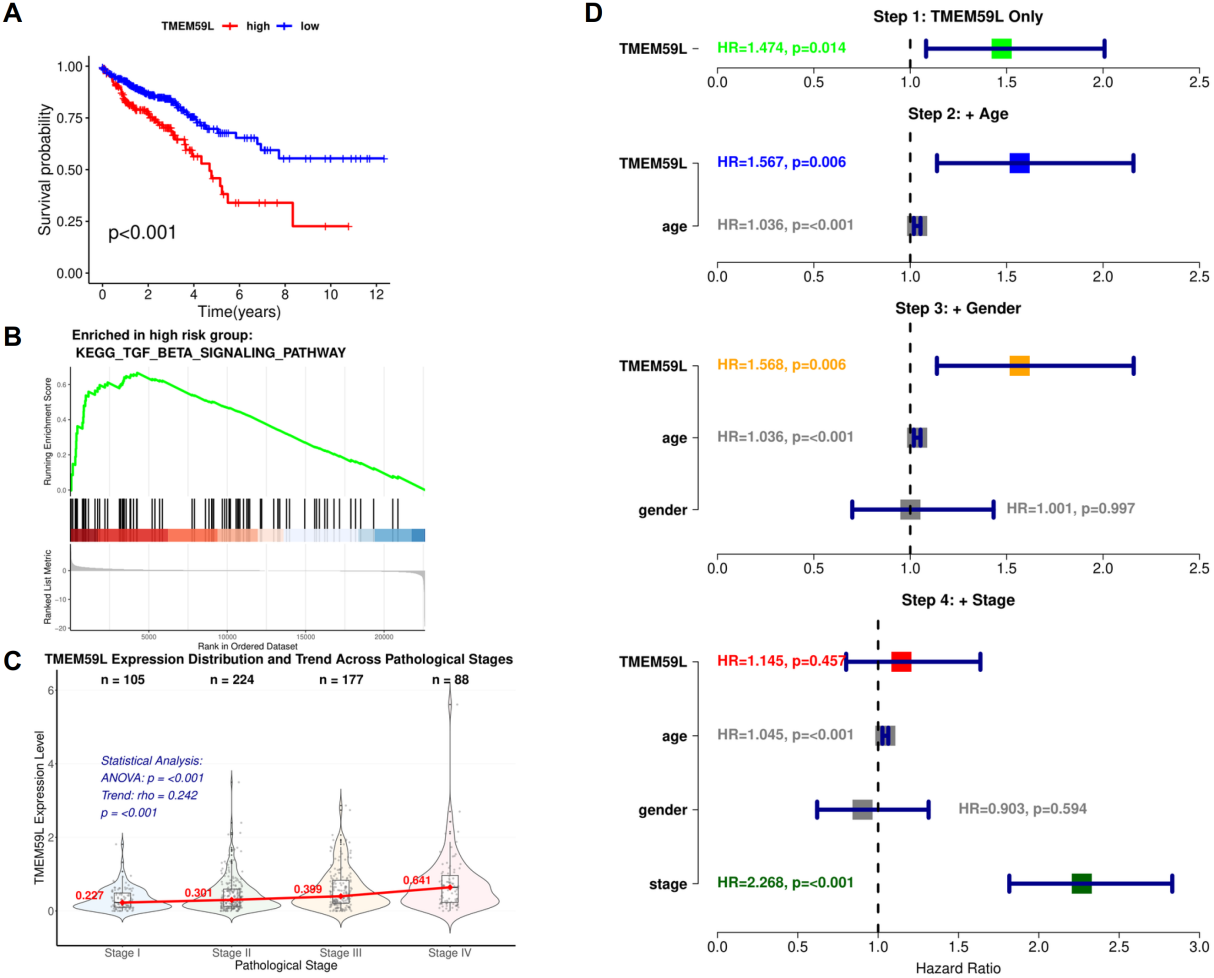
Bioinformatics analysis of TMEM59L. **(A)** Kaplan-Meier Survival Analysis of Colorectal Cancer Patients Stratified by TMEM59L Expression Levels. **(B)** GSEA Enrichment Plot of the KEGG TGF-β Signaling Pathway in TMEM59L High-Expression Group. **(C)** Violin Plot of TMEM59L Expression Distribution Across Pathological Stages (I-IV) with Trend Analysis. **(D)** Forest Plot of Stepwise Cox Regression Analysis Adjusting for Age, Gender, and Pathological Stage.

Based on the Kaplan-Meier survival analysis, patients with high TMEM59L expression showed significantly poorer survival rates compared to those with low TMEM59L expression (**Figure 1A**). The GSEA results for the high TMEM59L expression group indicate significant enrichment in the KEGG TGF-β signaling pathway (**Figure 1B**). Analysis of TMEM59L expression across pathological stages revealed a significant increasing trend (Spearman rho = 0.242, p < 0.001), with mean expression levels progressively rising from Stage I (0.321 ± 0.304) to Stage IV (0.743 ± 0.777) (**Figure 1C**). Multivariable Cox regression analysis showed that while TMEM59L was significantly associated with survival in univariate analysis (HR = 1.474, 95% CI: 1.082-2.008, p = 0.014). TMEM59L remained significant after adjusting for age and gender but lost significance after stage adjustment (HR = 1.145, p = 0.457), suggesting the prognostic effect may be mediated by tumor stage (**Figure 1D**).

### Knockdown of TMEM59L inhibits metastasis of colorectal cancer cells *in vivo* and *in vitro*

**Figure 2:**
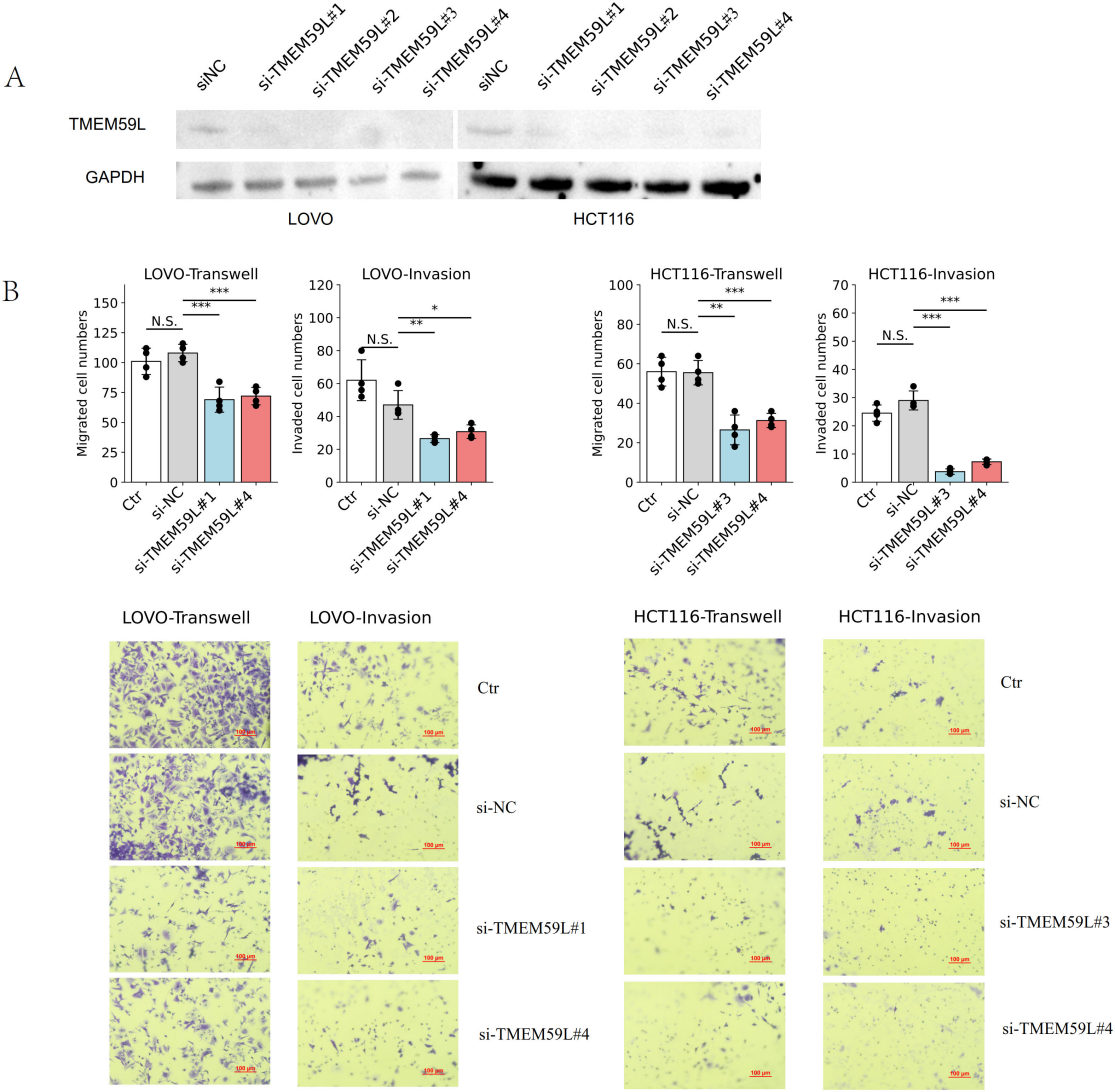
Effects of TMEM59L on cell motility *in vitro.***(A)** Validation of the efficiency of TMEM59L knockdown after transfection with different siRNAs for 48 h **(B)** TMEM59L knockdown inhibited cell motility. After siRNA transfection for 48 h, cells were subjected to migration or invasion assay for 24 h. *p < 0.05; **p < 0.01; ***p < 0.001.

**Figure 3:**
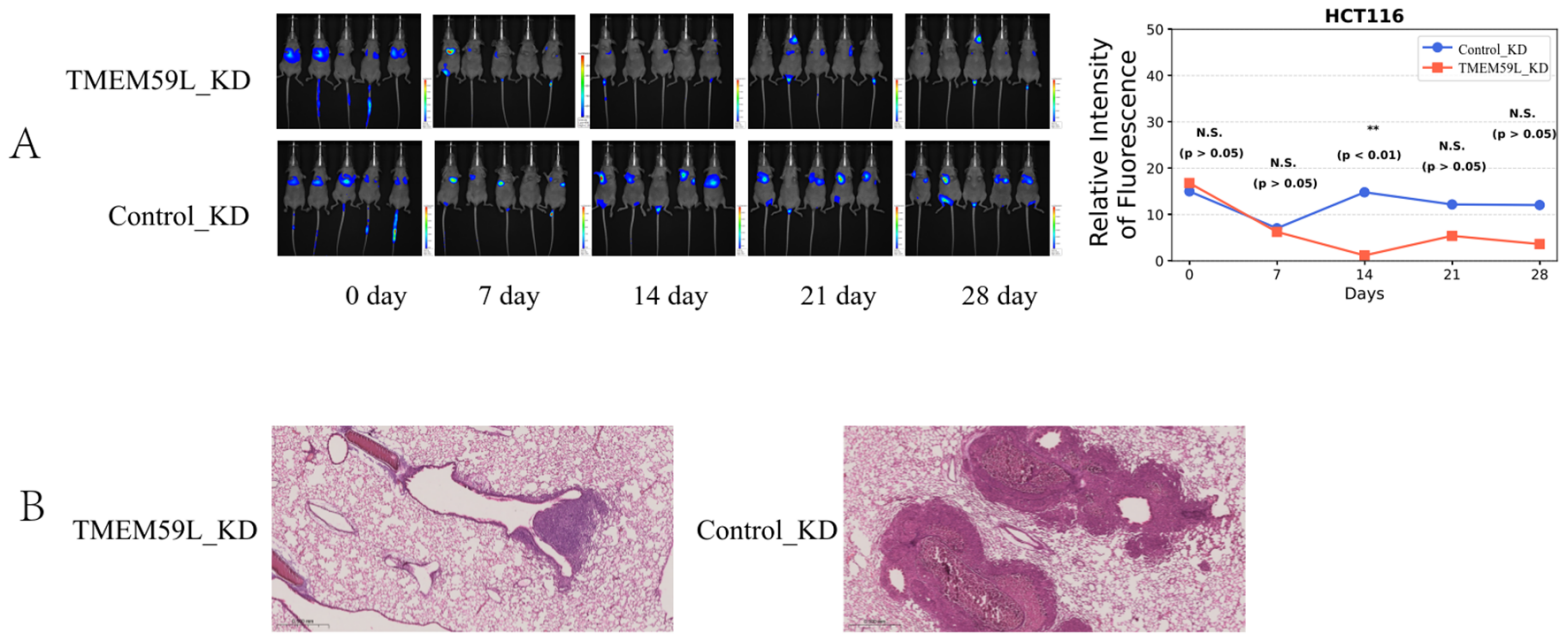
Effects of TMEM59L on cell motility *in vivo.***(A)** Live bioluminescence imaging of nude mice following HCT116 cell injection. Bioluminescence signals were obtained using an IVScope 8200 imaging system at 0, 7, 14, 21, and 28 days post-treatment. Relative intensity of fluorescence in two groups of nude mice. **(B)** Hematoxylin-eosin staining of the lung tissues of mice. **p < 0.01.

Quantitative PCR validation confirmed successful generation of stable cell lines. TMEM59L_KD showed 92% reduction in mRNA expression (0.08-fold relative to Control_KD), while TMEM59L_OE demonstrated 384-fold increase in TMEM59L mRNA levels compared to Control_OE.We firstly treated two colorectal cancer cell lines HCT116 and LoVo with siRNA-TMEM59L with four different sites small interfering RNA and the siRNA interference effect was effective (**Figure 2A**). The complete, unprocessed Western blot images are provided in **Supplementary Files 1–4**. In the following experiment, we selected two siRNAs for each cell line for further experiments. We then performed migration and invasion assays. Knockdown of TMEM59L in colorectal cancer cells inhibited the migration and invasion abilities of both LoVo and HCT116 cells (**Figure 2B**). To evaluate the influence of TMEM59L knocked down on cancer cell motility *in vivo*, we injected TMEM59L_KD cells into caudal vein of nude mice and fluorescence was observed continuously for 4 weeks, and fluorescence photographs were taken once a week. TMEM59L_KD cells had poor transfer capacity. At the beginning, the fluorescent intensity of nude mice in both groups was similar, and then the fluorescent intensity was detected every seven days. The fluorescent intensity of the control group gradually became stronger than that of the experimental group, and by day 14, the difference was statistically significant (**Figure 3A**). The Hematoxylin-eosin staining of sectioned lung tissues also showed that HCT116-luc#shTMEM59L cells groups had less metastases (**Figure 3B**). These results suggest that TMEM59L knocked down inhibit motility of a subset of colorectal cancer cells *in vitro* and *in vivo*.

### Effect of TMEM59L on cell growth and proliferation

**Figure 4:**
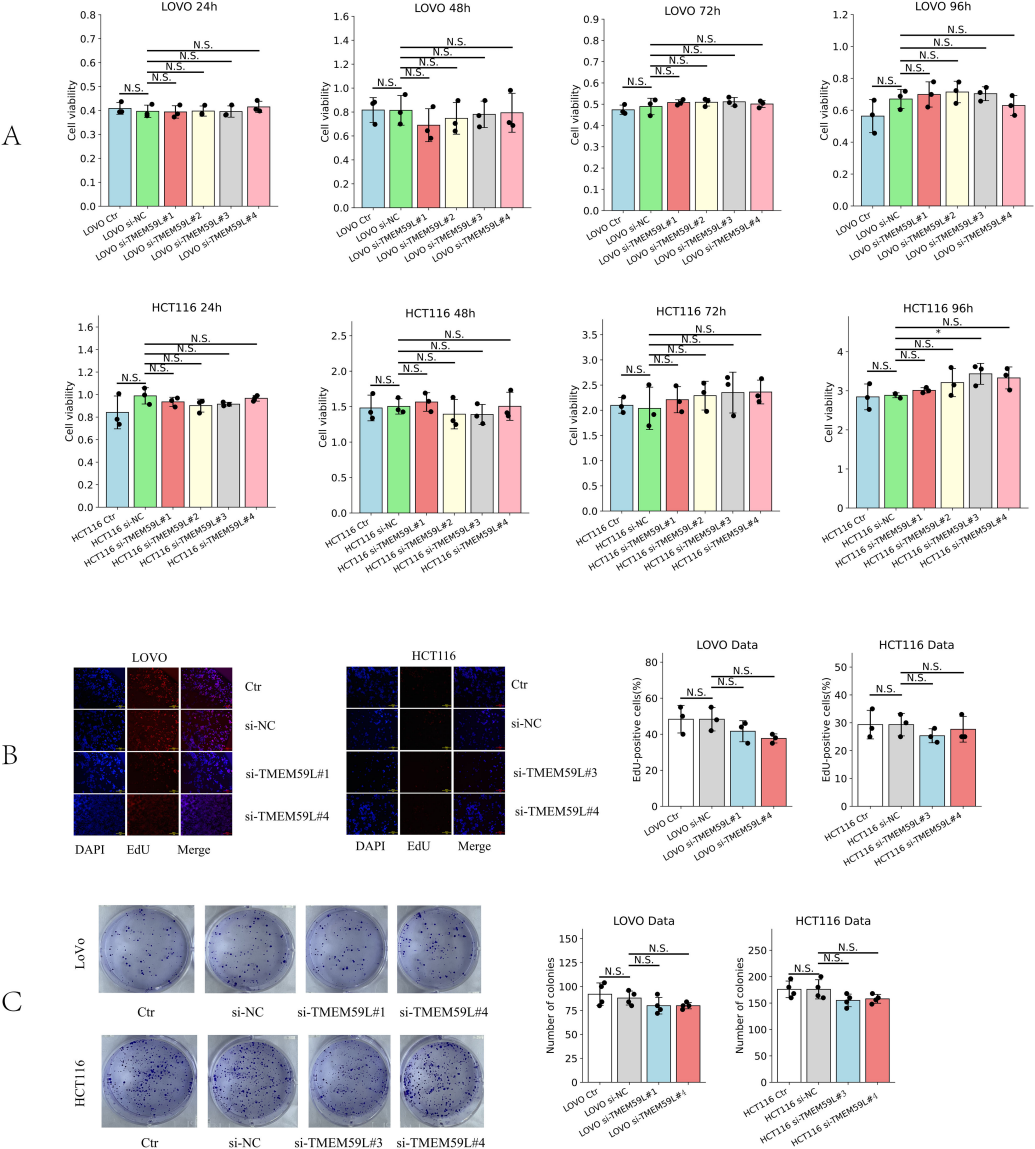
Effect of TMEM59L on cancer cell proliferation. **(A)** Effect of TMEM59L on cancer cell proliferation detected by CCK-8 assay. **(B)** Effect of TMEM59L on cancer cell proliferation detected by EdU incorporation assays. **(C)** HCT116 and LoVo cells were incubated with siRNA-TMEM59L or NC for 10 days, and the number of colonies were calculated.

To investigate whether TMEM59L could influence cell growth in HCT116 and LoVo cells, si-TMEM59L was used to treat HCT116 and LoVo cells, and detect the cell viability at different time points (24, 48, 72 and 96 h) by CCK-8 assay. The results show that si-TMEM59L did not have a significant positive or negative influence on cell viability in most groups of HCT116 and LoVo cells. Only in HCT116 cells treated with si-TMEM59L#3 at 96 hours was an increase in cell viability observed. (**Figure 4A**). Furthermore, the influence of cell growth by TMEM59L was tested by cloning formation and EdU incorporation assays. After si-TMEM59L treatment, the colony numbers showed no statistically significant difference (N.S.) across all groups in both LoVo and HCT116 cells (**Figure 4B**). Similarly, after si-TMEM59L treatment, the percentage of EdU-positive cells, which indicates actively proliferating cells, did not exhibit any significant changes in either LoVo or HCT116 cells (**Figure 4C**).

### TGF-β and Smad signaling pathway mediates TMEM59L expression

**Figure 5:**
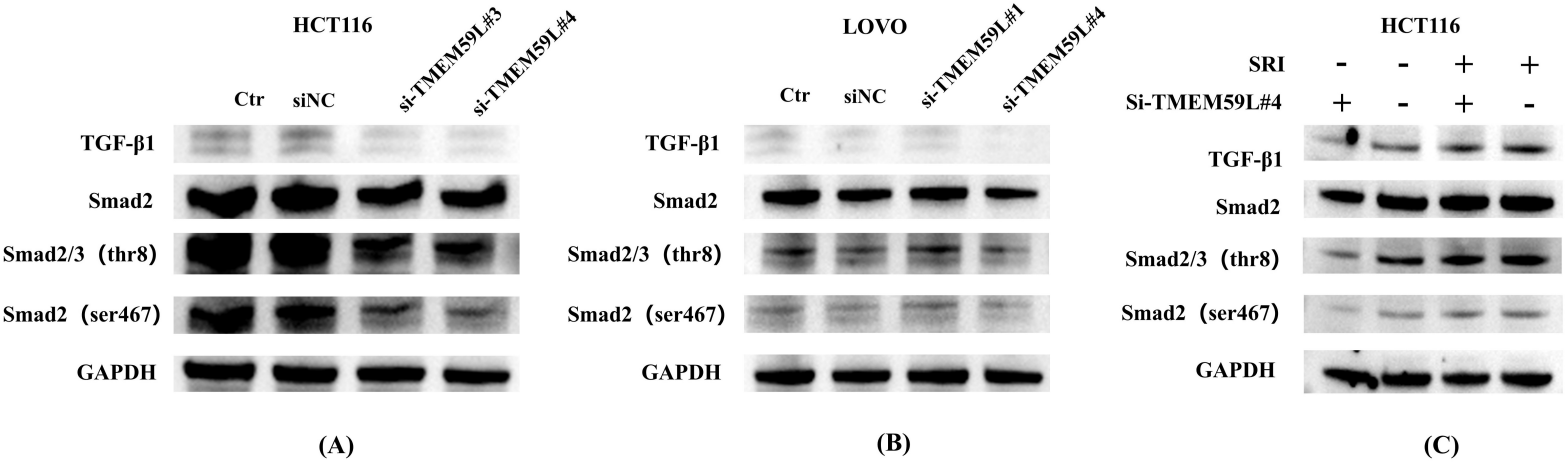
TGF-β pathway mediates TMEM59L expression *in vitro*. **(A)** Western blot analysis of Smad phosphorylation and TGF-β expression in HCT116 cells transfected with TMEM59L-specific siRNA for 48 hours. The same GAPDH loading control is shown for all groups in panel **(A, B)** Western blot analysis of Smad phosphorylation and TGF-β expression in LoVo cells transfected with TMEM59L-specific siRNA for 48 hours. The same GAPDH loading control is shown for all groups in panel **(B, C)** Western blot analysis of Smad phosphorylation and TGF-β expression in HCT116 cells pretreated with SRI-011381 for 1 hour, followed by TMEM59L-specific siRNA treatment for 48 hours. The same GAPDH loading control is shown for all groups in panel **(C)**.

**Figure 6:**
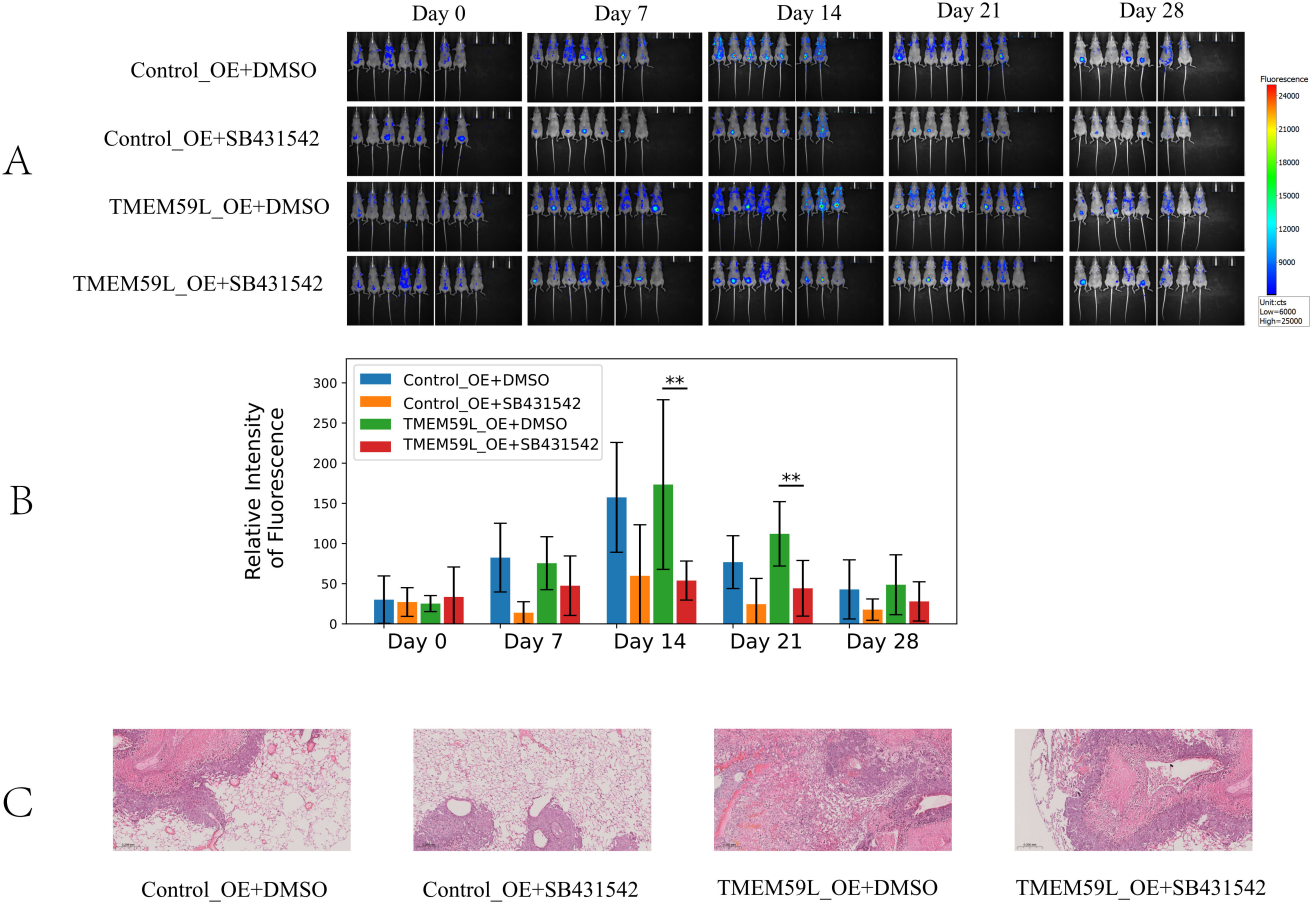
TGF-β pathway mediates TMEM59L expression *in vivo.***(A)** Live bioluminescence imaging of nude mice following HCT116 cell injection. Bioluminescence signals were obtained using an IVScope 8200 imaging system at 0, 7, 14, 21, and 28 days post-treatment. Relative intensity of fluorescence in two groups of nude mice. **(B)** Relative intensity of fluorescence in two groups of nude mice. **(C)** Hematoxylin-eosin staining of the lung tissues of mice. **p < 0.01.

Data analysis prompted TMEM59L exerting a key influence in cancer development may associated TGF-β signaling (17). Our enrichment analysis of the latest TCGA data reached the same conclusion, so we conducted experimental exploratory verification. After siRNA TMEM59L interference, we found that the TGF-β signaling pathway may play a vital role. TGF-β pathway is involved in activation of Smad signaling pathway (20). We examined the roles of TGF-β and Smad signaling pathway. Treatment with interfering the expression of TMEM59L strongly inhibite TGF-β and inhibited phosphorylation levels of Smad2/3 in HCT116 cells (**Figure 5A**), but this phenomenon was not obvious in LoVo cells (**Figure 5B**). The complete, unprocessed Western blot images are provided in **Supplementary Files 5–9**. SRI-011381 is TGF-β agonist, in HCT116 cells, after treating in SRI-011381, TMEM59L interference induced inhibited phosphorylation levels of Smad2/3 was abrogated (**Figure 5C**). The complete, unprocessed Western blot images are provided in **Supplementary Files 10–14**. In animal experiments, To evaluate the impact of TMEM59L overexpression (TMEM59L_OE) on *in vivo* motility and the relationship between SB431542 and TMEM59L, we divided mice into four groups: Ctr+DMSO, Ctr+SB431542, TMEM59L_OE+DMSO, and TMEM59L_OE+SB431542. Fluorescence images were taken weekly and observed continuously for 4 weeks. The study found that, starting from day 14, the fluorescence intensity in the TMEM59L_OE+DMSO group was higher than that in the Ctr+DMSO group. Furthermore, starting from day 7, the fluorescence intensity in the TMEM59L_OE+SB431542 group was reduced compared to the TMEM59L_OE+DMSO group (**Figure 6A**). The fluorescence intensity in the TMEM59L_OE+SB431542 group was significantly reduced compared to the TMEM59L_OE+DMSO group on day 14 and day 21 (p < 0.05) (**Figure 6B**). Hematoxylin-eosin staining of sectioned lung tissues also showed that the SB431542 TMEM59L_OE+SB431542 group had fewer metastatic lesions compared to the TMEM59L_OE+DMSO group (**Figure 6C**).

## Discussion

TMEM59L first appeared in a 1999 article, which reported the discovery of a gene encoding a novel protein, brain-specific membrane-associated protein (BSMAP), now known as TMEM59L. Northern blot analysis showed that BSMAP mRNA is preferentially and highly expressed in the brain, with the protein predicted to be a type I membrane glycoprotein, potentially involved in the functions of the central nervous system (13). In 2014, Aoki et al. (21) reported that TMEM59L is a gene associated with axon growth and is downstream of Islet2a, playing a role in the development of sensory neurons. In 2016, Kobayashi et al. (22) reported that knockdown of TMEM59L significantly reduced insulin secretion from MIN6c4 cells under glucose and/or KCl stimulation, but did not significantly alter the cellular insulin content. However, overexpression of TMEM59L increased insulin secretion. In 2017, Zheng et al. (14) reported that TMEM59L is primarily localized to the Golgi and endosomes. It can interact with autophagy-related proteins ATG5 and ATG16L1, and its overexpression triggers autophagy, promoting caspase-dependent cell apoptosis. Knockdown of TMEM59L reduces anxiety and depression in mice. In 2020, Rutledge et al (23). reported that TMEM59L, along with other genes such as Hs3st3a1 and Hs3st3b1, is involved in the ureteric branching program, which plays a role in the branching morphogenesis of the ureter during kidney development. In 2024, Yuan et al (15). reported that TMEM59L is one of the neuronal marker genes and is highly expressed in differentiated neuronal cell clusters, suggesting that it may be involved in the differentiation process of adipose-derived stromal cells induced into neurons, suggesting its potential as a marker for neurodevelopmental processes.

The relationship between TMEM59L and cancers was first reported in 2022. In that year, Kołat et al. (24) reported that TMEM59L may act as a target of the AP-2 transcription factor and be involved in cancer progression. In the same year, a study by Chang Shi et al. (17) discovered that high TMEM59L expression is associated with shorter survival in patients with various cancers, while higher TMEM59L methylation levels correlate with longer survival. In 2023, Yang et al (18) reported that TMEM59L is a key marker gene for predicting lymph node metastasis in CRC patients and further confirmed that its high expression is closely associated with shortened overall survival in CRC patients. In 2024, Liu et al (19). also found an association between high TMEM59L expression and shortened overall survival in CRC patients.

Our study demonstrates that high TMEM59L expression in CRC is linked to poor survival and significant enrichment of the TGF-β signaling pathway. Kaplan-Meier analysis revealed that patients with high TMEM59L expression had worse survival compared to those with low expression, supporting its role in cancer progression. GSEA further confirmed enrichment of the TGF-β pathway in the high TMEM59L group, suggesting that TMEM59L may influence tumor biology and contribute to CRC metastasis via this pathway.

Cell migration and invasion are key processes for tumor cells to acquire metastatic potential. In *in vitro* cell experiments, enhanced migration and invasion are typically associated with epithelial-mesenchymal transition (EMT) (25, 26), cell adhesion and detachment (27), activation of specific signaling pathways (such as TGF-β signaling pathway) (28), and immune microenvironment modulation (29). This study is the first to investigate the association between TMEM59L and cancers through cell and animal experiments. We found that knockdown of TMEM59L in HCT116 and LoVo cells significantly inhibited their migration and invasion, both *in vitro* and *in vivo*. Regarding cell proliferation and growth, knockdown of TMEM59L did not significantly affect cell viability, colony formation, or EdU incorporation assays in most cell lines, suggesting that TMEM59L may not directly regulate CRC cell growth under these experimental conditions. However, we did observe an increase in cell viability in HCT116 cells 96 hours after treatment with si-TMEM59L#3, suggesting that TMEM59L may play a role in suppressing tumor cell viability. The selective effect of TMEM59L on cell migration and invasion, with minimal impact on proliferation, represents an interesting functional profile that warrants further investigation.

In the early stages of cancer, TGF-β protein exerts a cancer-suppressive effect by inducing cell cycle arrest and apoptosis. During cancer progression, cancer cells gradually develop resistance and begin to secrete TGF-β on their own (30). The aberrant activation of the TGF-β signaling pathway can promote tumor cell migration, invasion, and EMT, as well as immune evasion, thereby driving tumor aggressiveness and metastasis (31). We found that TMEM59L knockdown significantly inhibited TGF-β/Smad signaling. Treatment with the TGF-β agonist SRI-011381 reversed the inhibitory effects of TMEM59L knockdown on Smad phosphorylation, suggesting that TMEM59L regulates metastasis via the TGF-β signaling pathway. *In vivo*, TMEM59L overexpression significantly promoted metastasis, as indicated by increased fluorescence intensity. Treatment with the TGF-β receptor inhibitor SB431542 reduced metastatic potential in the overexpression groups as demonstrated by decreased fluorescence intensity, further confirming the involvement of the TGF-β pathway in TMEM59L-mediated metastasis. Our study is the first to reveal the role of TMEM59L in colorectal cancer metastasis through animal and cell experiments. While our study demonstrates that TMEM59L modulates TGF-β signaling, the precise molecular mechanism remains to be elucidated. Previous studies have shown that TMEM59L is primarily localized to the Golgi and endosomes and can interact with autophagy-related proteins (14). Given this subcellular localization and our observation of concurrent reduction in TGF-β protein levels and Smad2/3 phosphorylation following TMEM59L knockdown, TMEM59L may regulate TGF-β signaling through intracellular trafficking or protein processing mechanisms. However, the specific molecular interactions require further investigation.

This study has several limitations. First, while we demonstrate that TMEM59L modulates TGF-β signaling, the precise molecular mechanism remains unclear—we cannot determine whether TMEM59L affects TGF-β transcription, protein secretion, or receptor signaling. Second, the tail vein injection model has inherent limitations as it bypasses the early stages of metastasis including local invasion and intravasation, therefore not fully recapitulating the complete spontaneous metastatic process that occurs in clinical settings. Third, the differential TGF-β pathway response to TMEM59L knockdown between HCT116 and LoVo cells remains unexplained and requires further investigation to determine the molecular basis for this cellular heterogeneity.

## Conclusions

In conclusion, our findings suggest that TMEM59L plays a pivotal role in CRC metastasis, potentially through the modulation of the TGF-β/Smad signaling pathway. TMEM59L promotes CRC metastasis by enhancing cell migration and invasion, with minimal impact on cell proliferation. These results highlight the potential of TMEM59L as a therapeutic target for inhibiting CRC metastasis, particularly through the modulation of the TGF-β/Smad pathway. Future research directions should include detailed mechanistic studies to elucidate how TMEM59L regulates TGF-β signaling, investigation of TMEM59L function in additional cancer models, and exploration of the molecular basis for cellular heterogeneity in pathway responses.

## Data Availability

The original contributions presented in the study are included in the article/**Supplementary Material**. Further inquiries can be directed to the corresponding author.
