# Supplementary figures and images for: The role of TMEM59L in colorectal cancer progression and its interaction with the TGF-β/Smad pathway
Source: Front Oncol. 2025 Nov 25;15:1674849. doi: 10.3389/fonc.2025.1674849 (PMC12685656; doi:10.3389/fonc.2025.1674849)

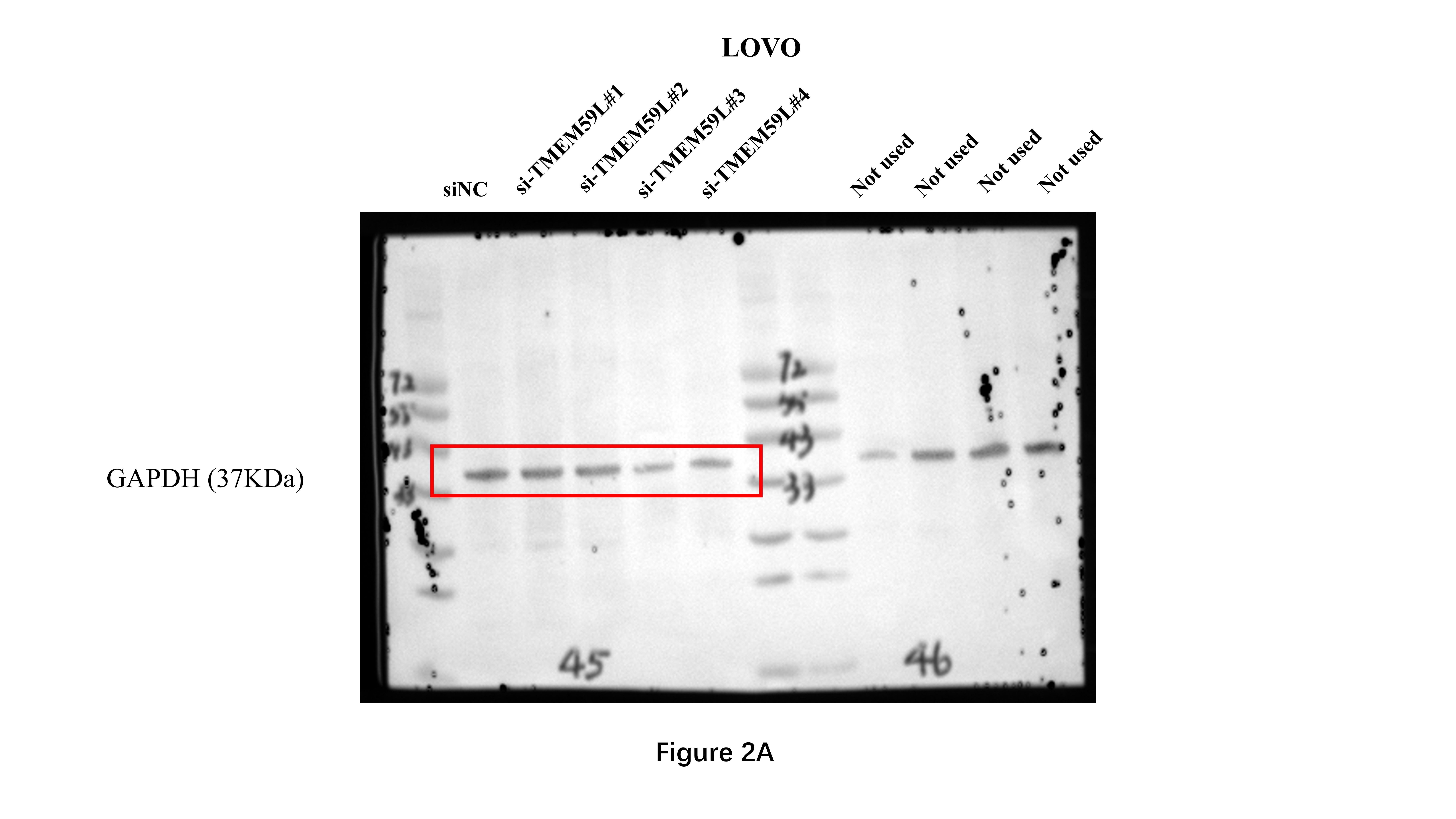

Supplement: Supplementary file 1 [file Image1.jpeg]

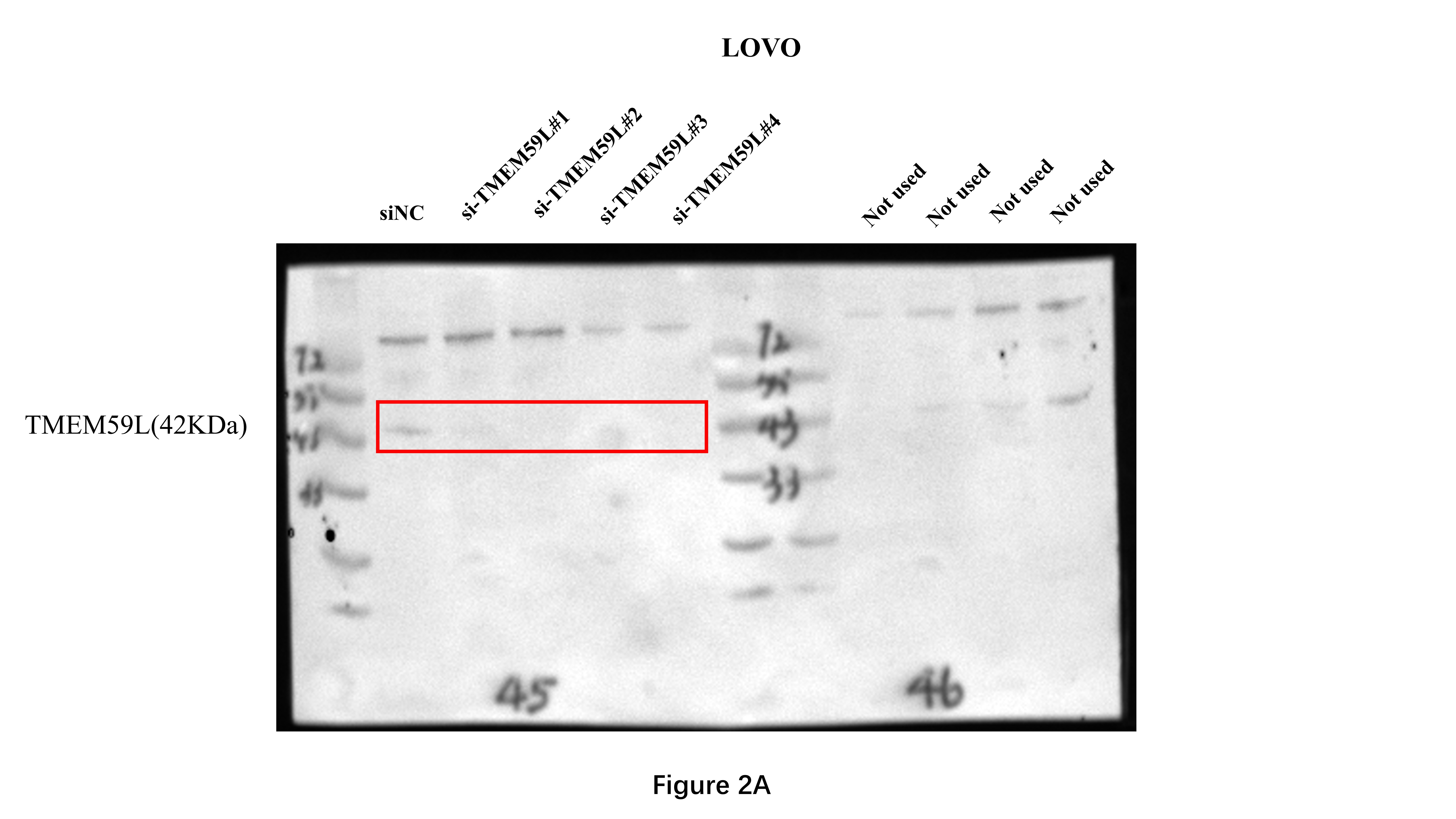

Supplement: Supplementary file 2 [file Image2.jpeg]

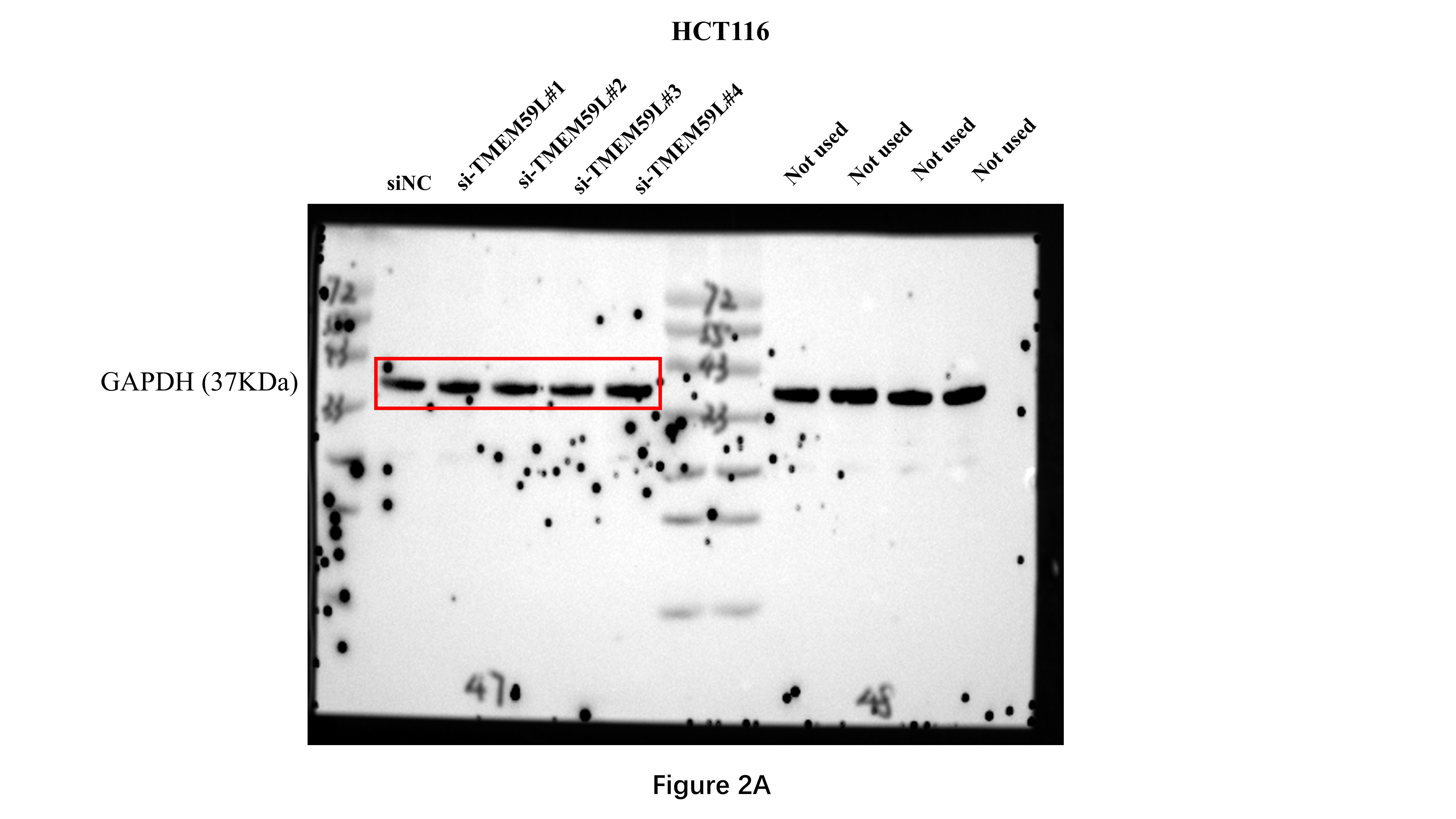

Supplement: Supplementary file 3 [file Image3.jpeg]

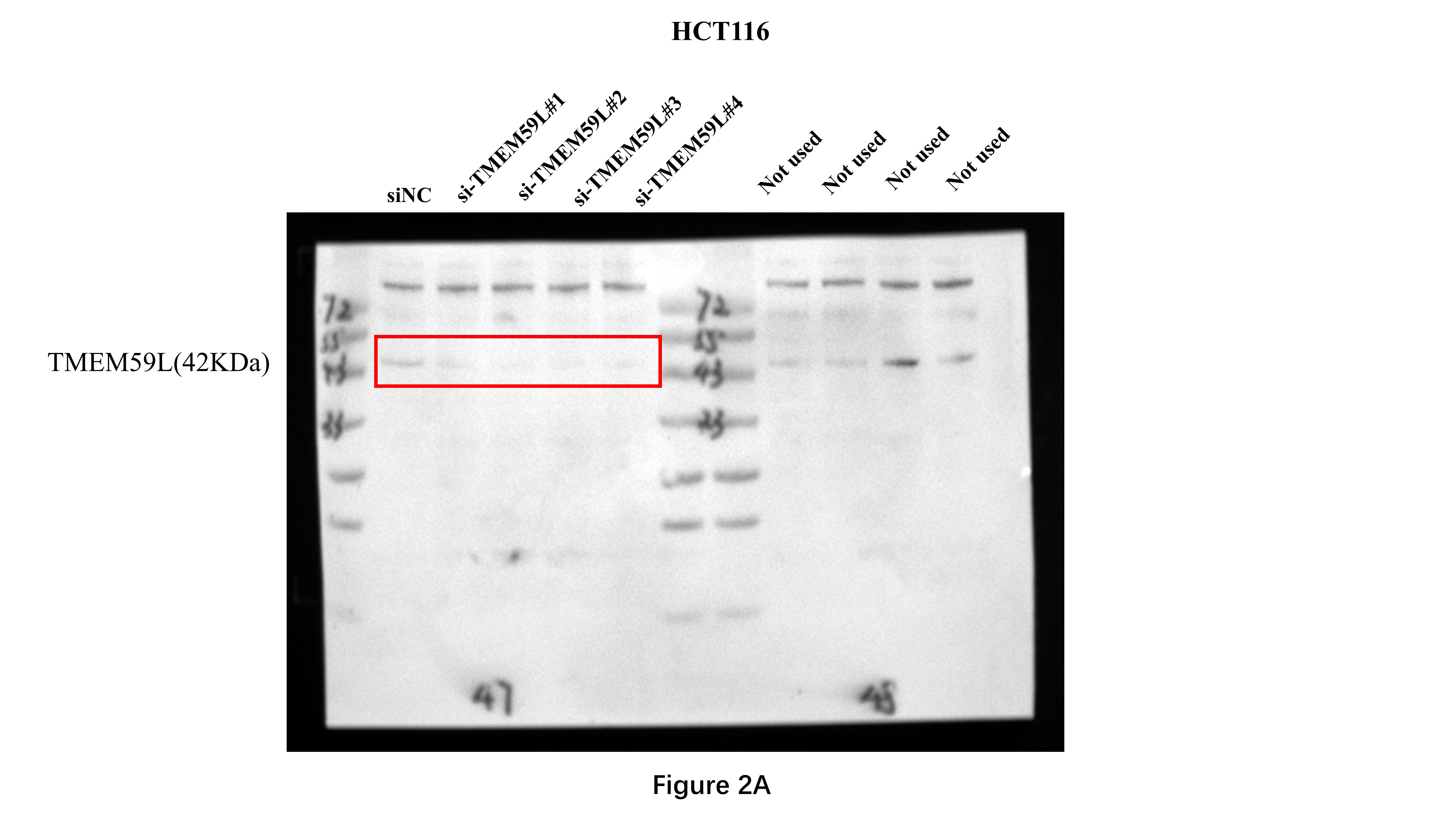

Supplement: Supplementary file 4 [file Image4.jpeg]

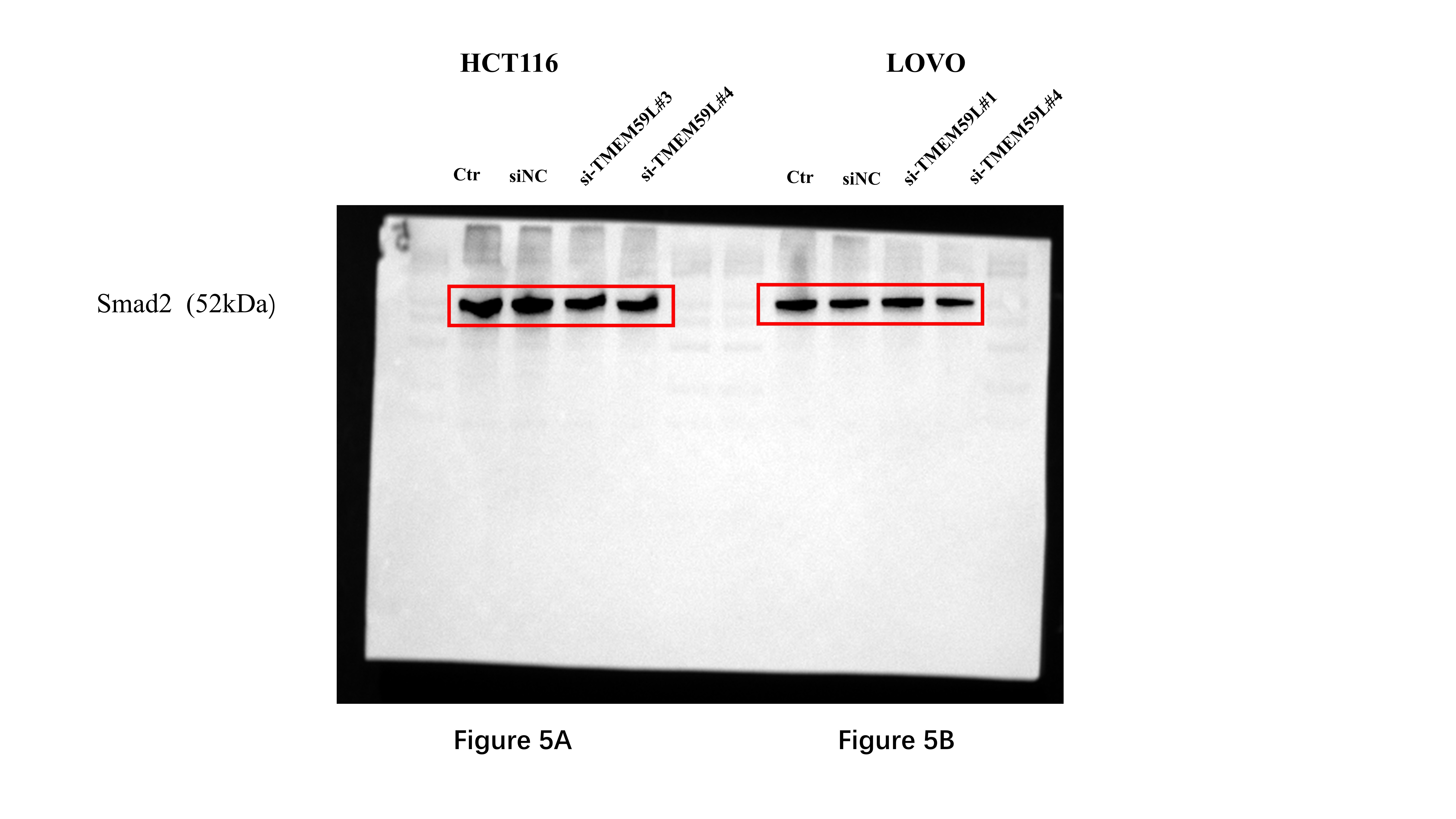

Supplement: Supplementary file 5 [file Image5.jpeg]

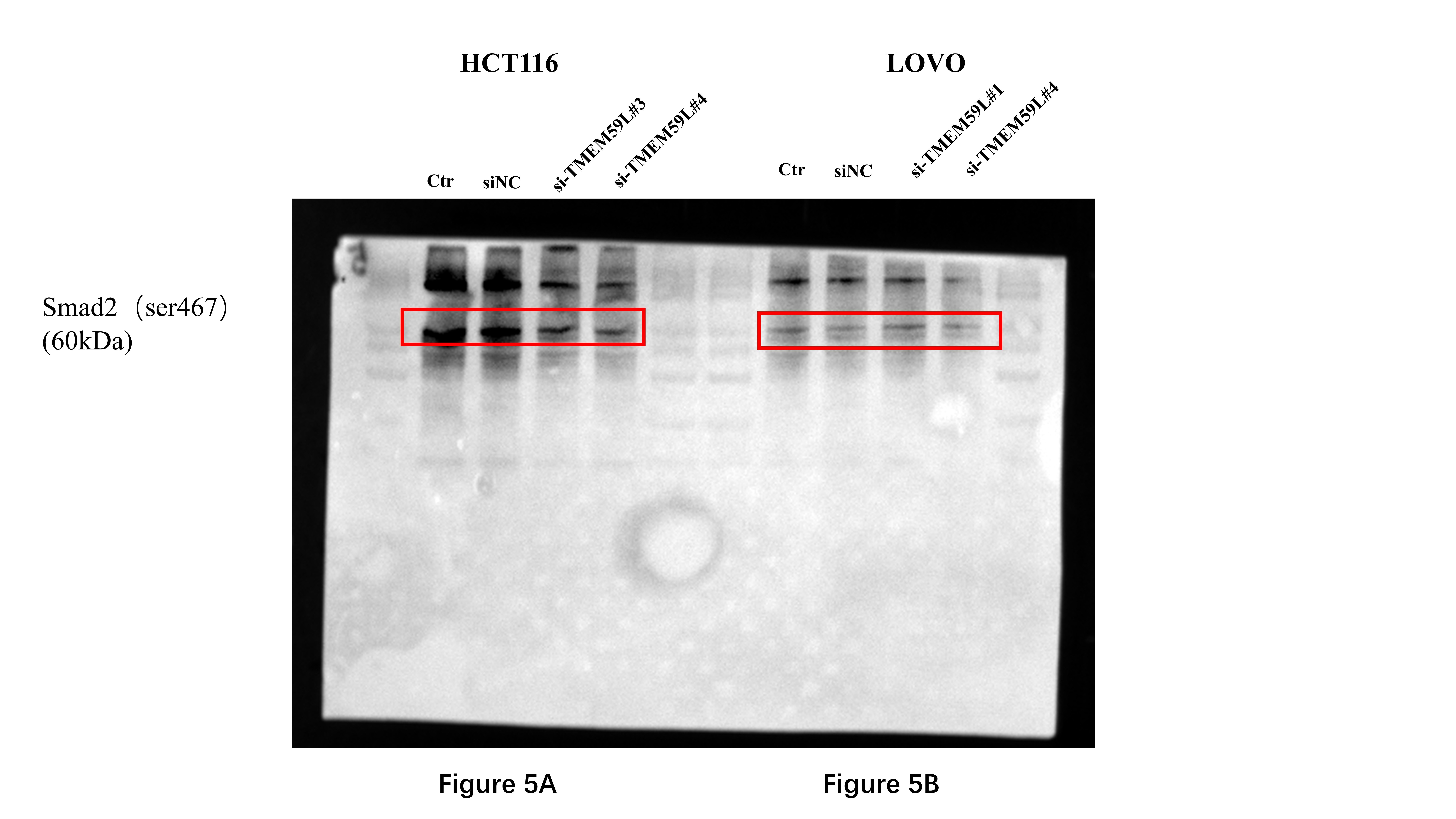

Supplement: Supplementary file 6 [file Image6.jpeg]

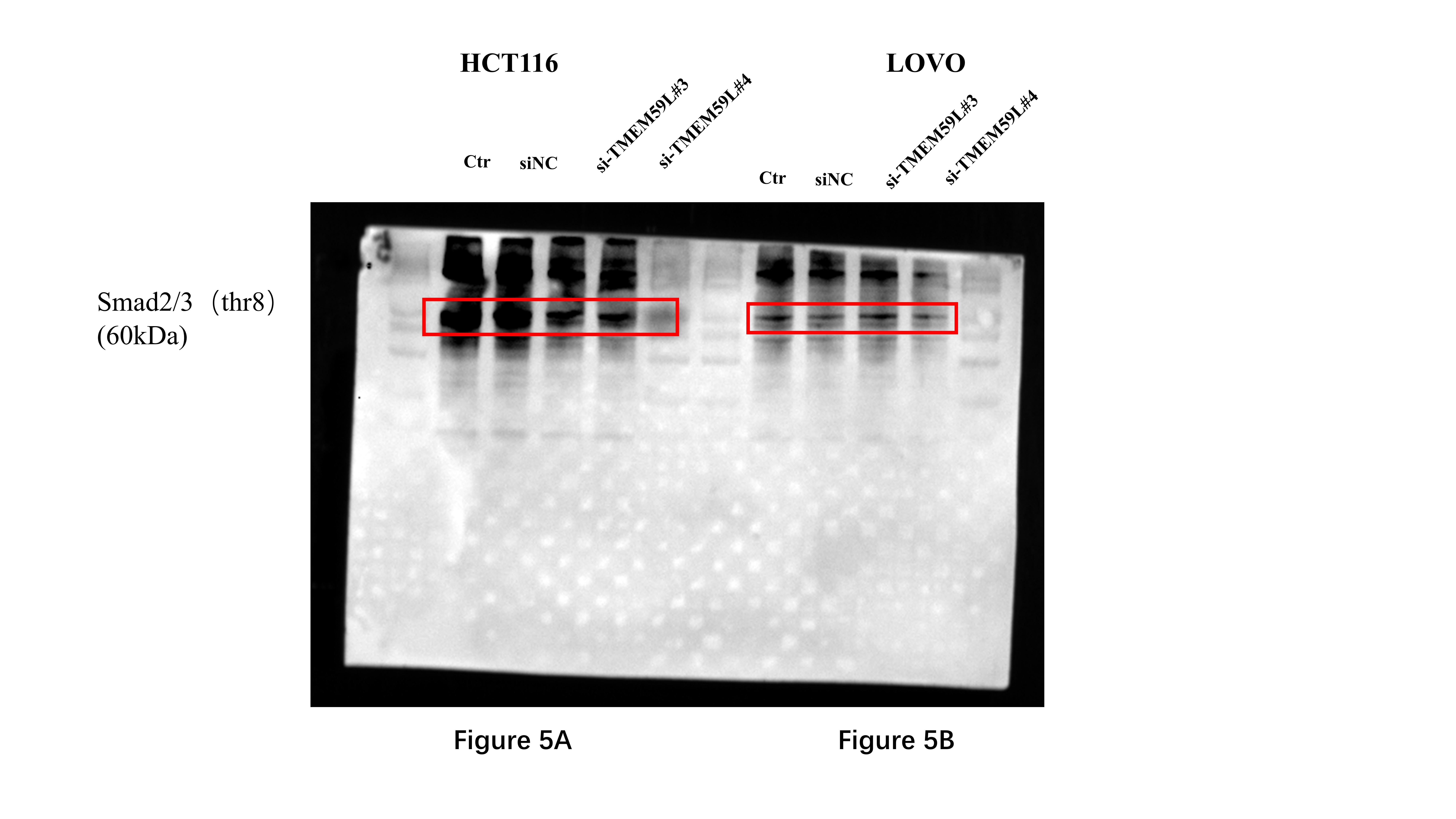

Supplement: Supplementary file 7 [file Image7.jpeg]

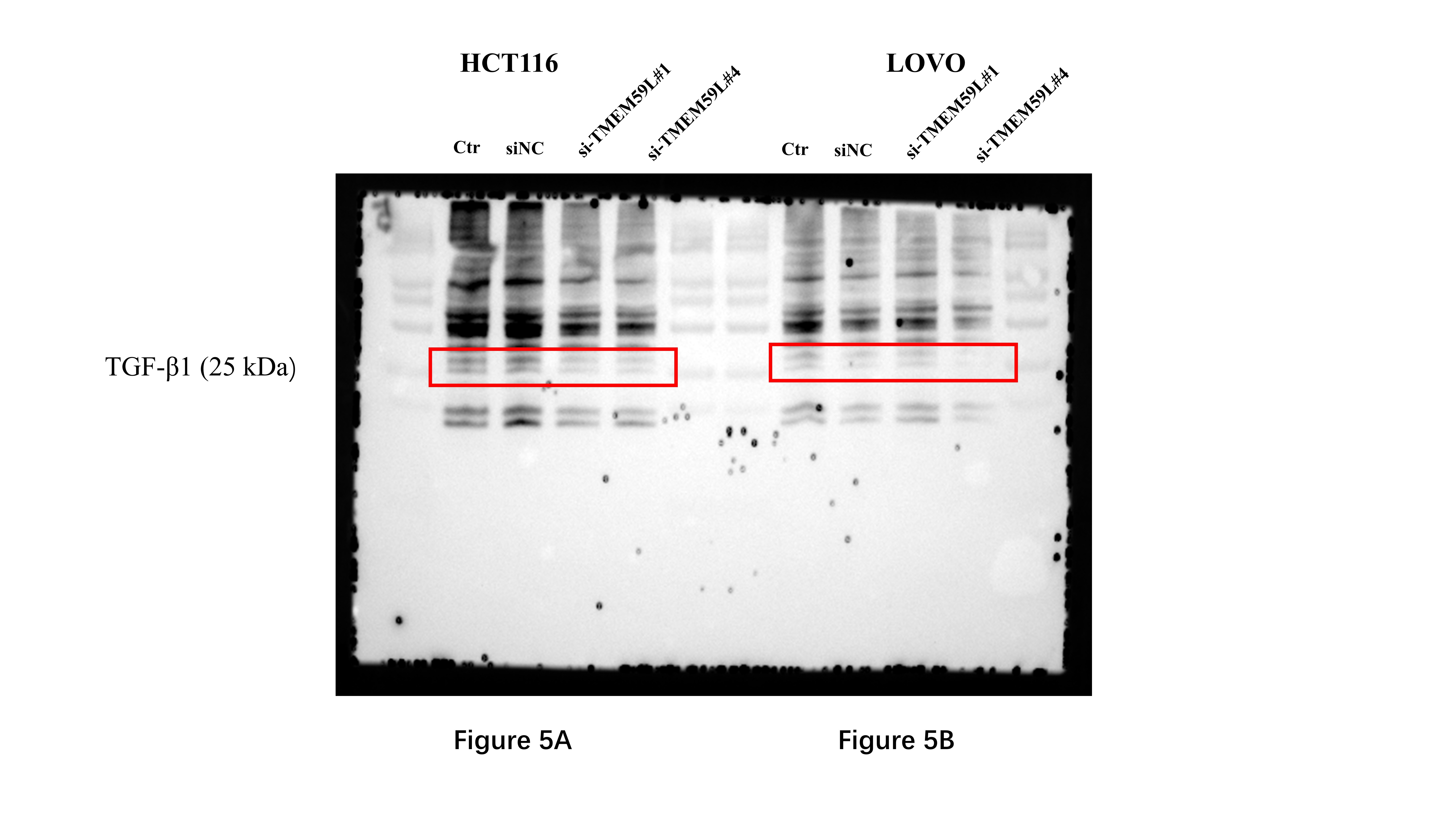

Supplement: Supplementary file 8 [file Image8.jpeg]

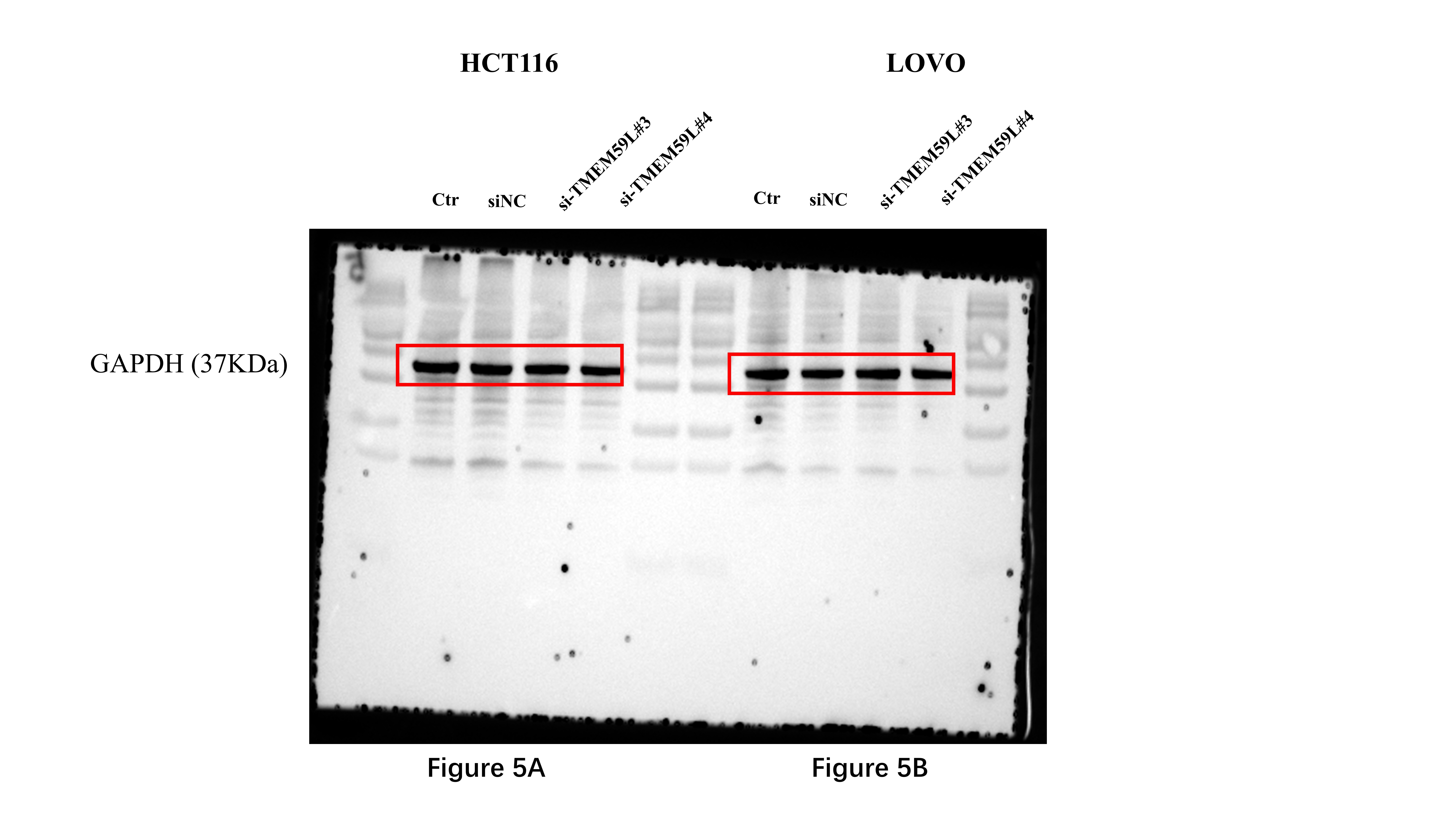

Supplement: Supplementary file 9 [file Image9.jpeg]

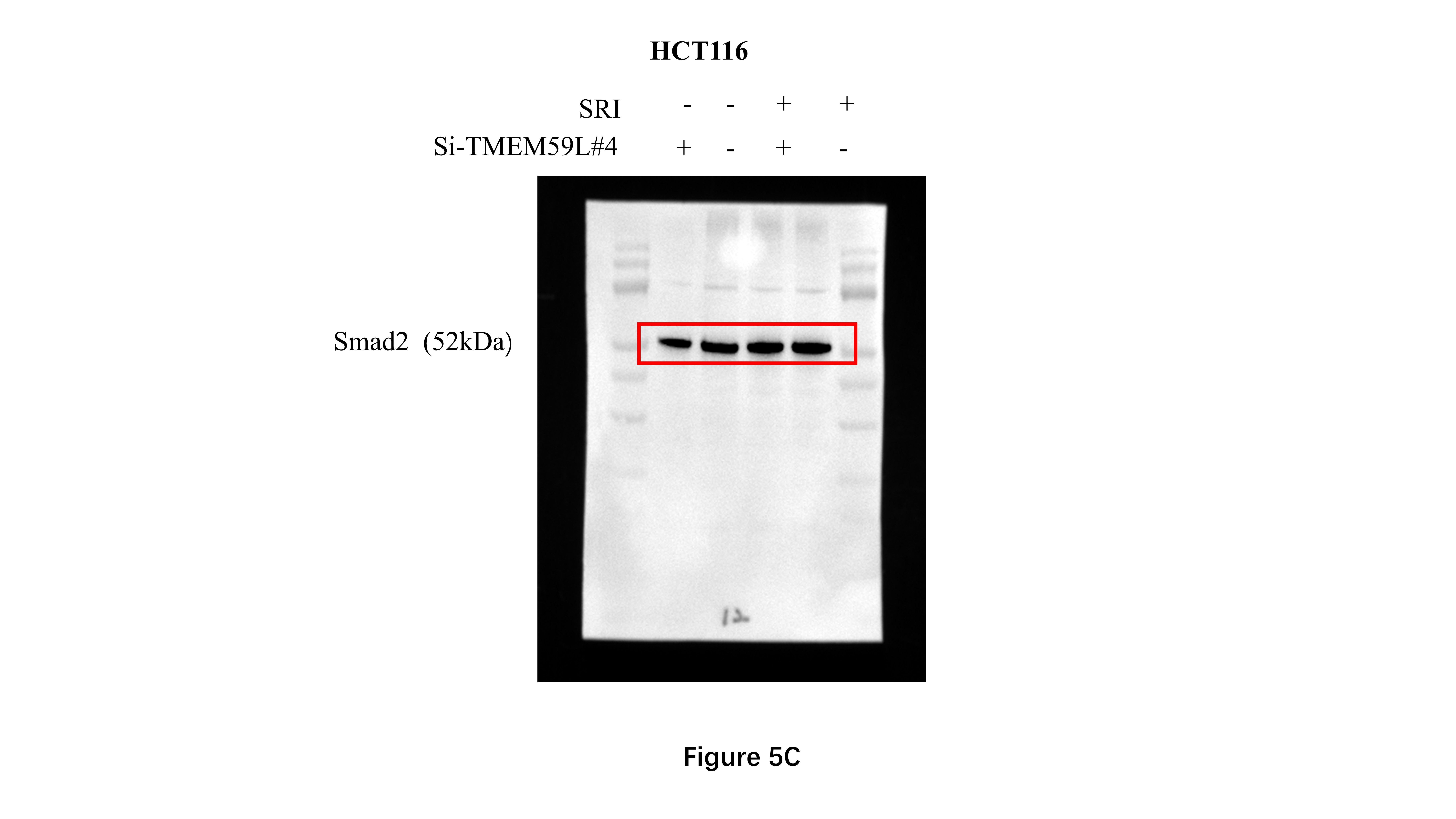

Supplement: Supplementary file 10 [file Image10.jpeg]

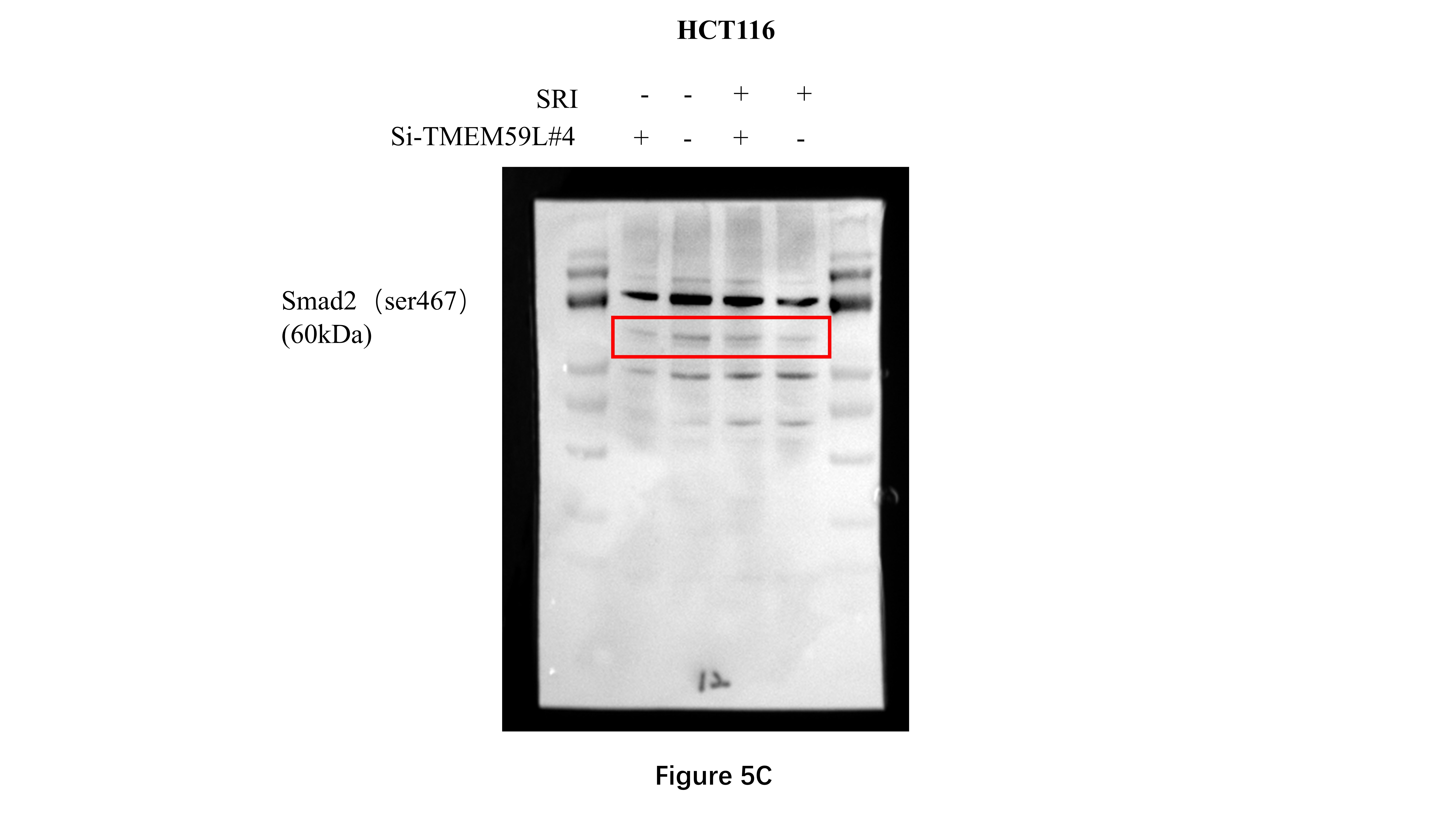

Supplement: Supplementary file 11 [file Image11.jpeg]

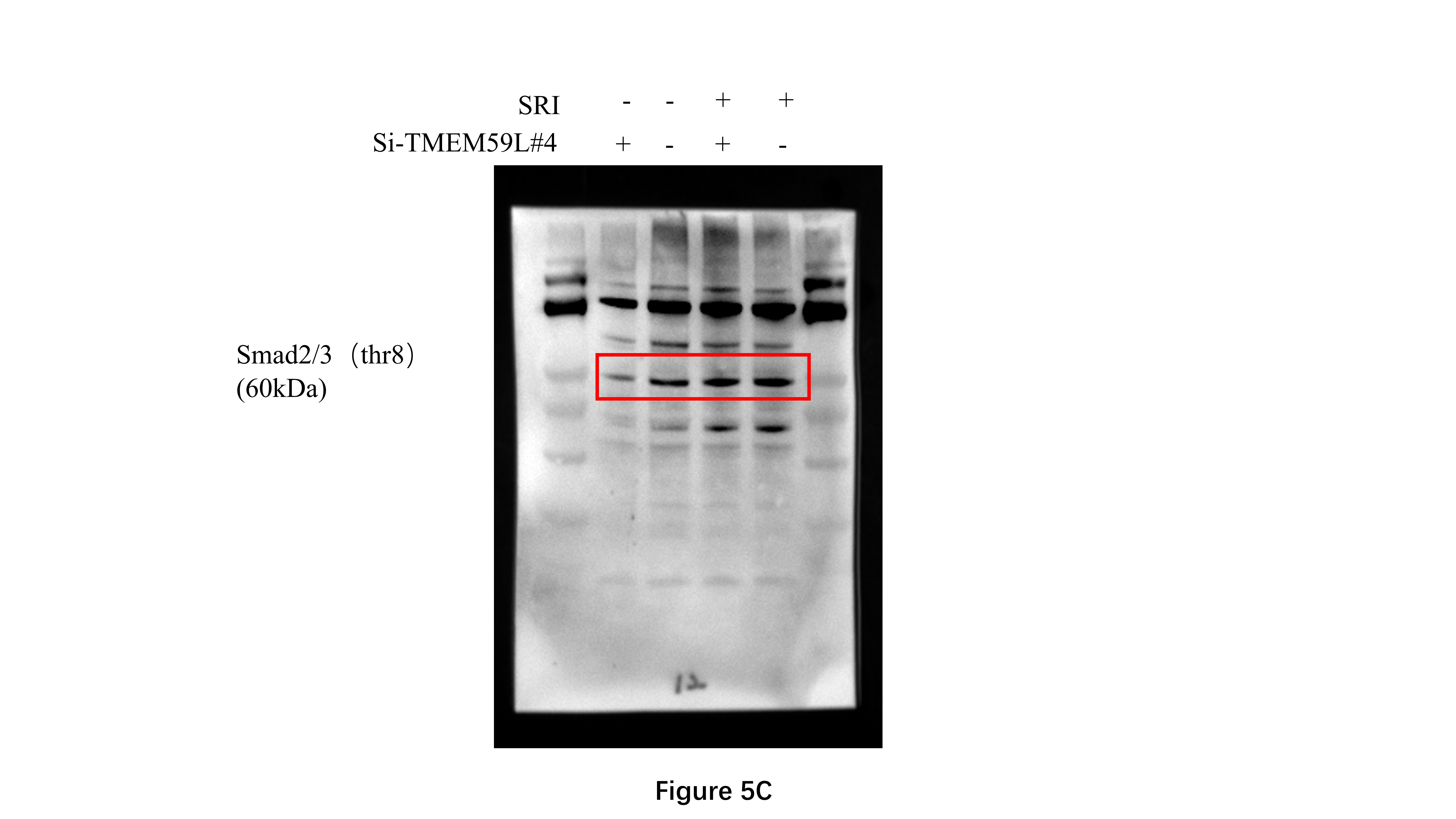

Supplement: Supplementary file 12 [file Image12.jpeg]

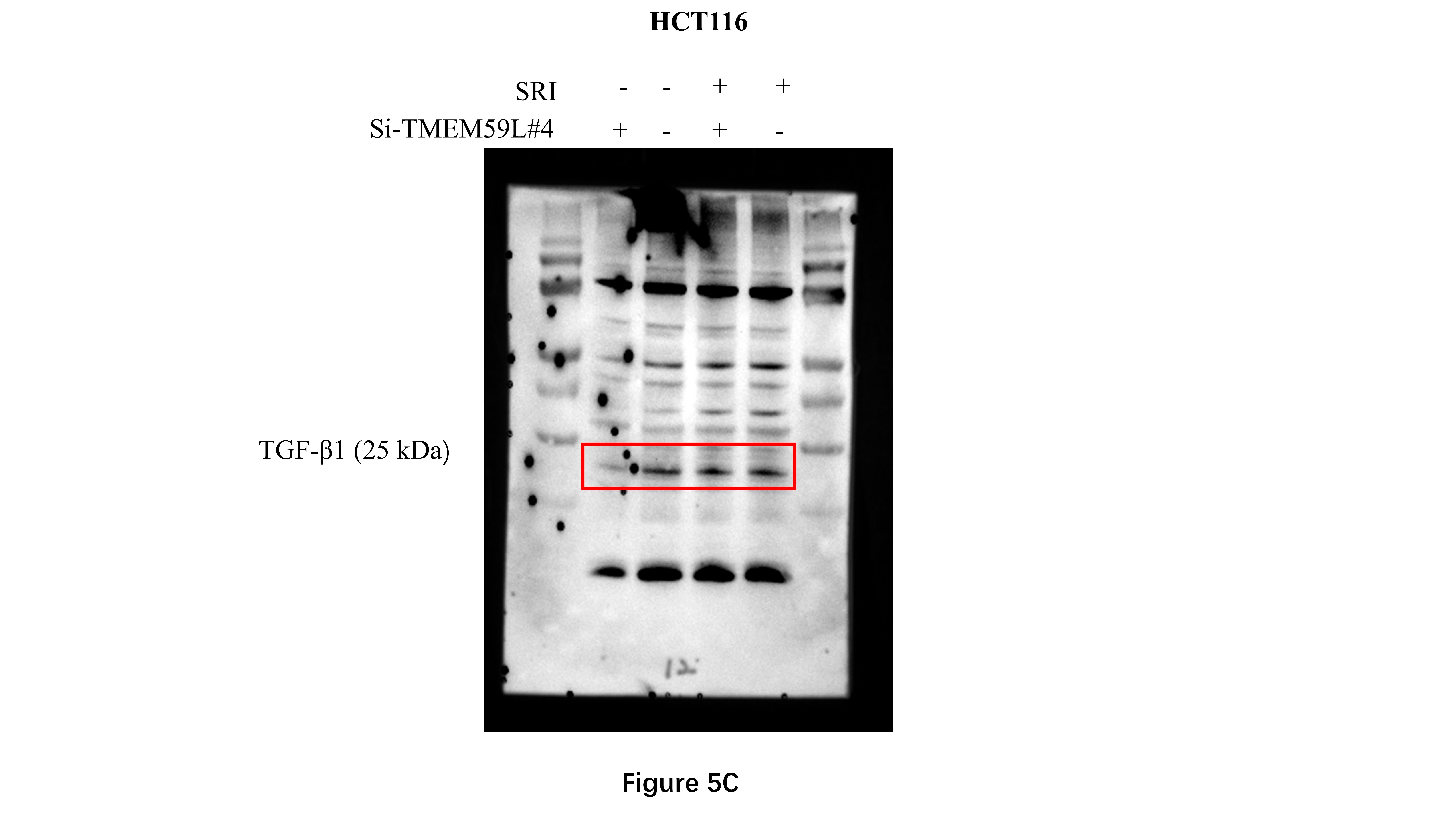

Supplement: Supplementary file 13 [file Image13.jpeg]

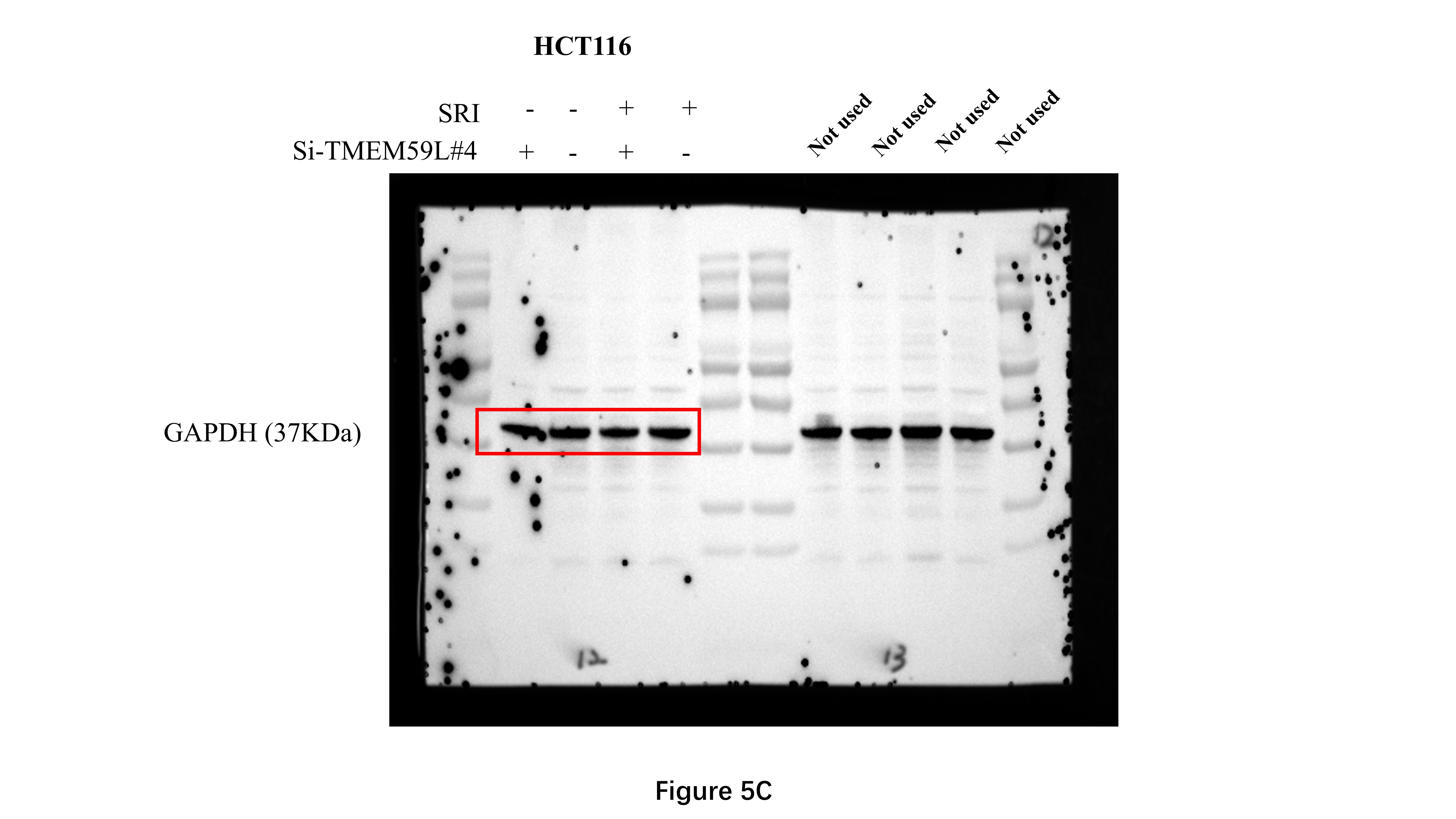

Supplement: Supplementary file 14 [file Image14.jpeg]
